# Development and Evaluation of a New Measles Detection Assay Using Real-Time RT-PCR

**DOI:** 10.3390/ijms26051801

**Published:** 2025-02-20

**Authors:** Vera A. Chayeb, Anna S. Dolgova, Margarita R. Popova, Nina V. Zheleznova, Svetlana A. Shirobokova, Anna V. Shabalina, Alena A. Sharova, Anna S. Gladkikh, Anastasia Yu. Antipova, Anastasiia D. Kirichenko, Edward S. Ramsay, Vladimir G. Dedkov

**Affiliations:** 1 Saint-Petersburg Pasteur Institute, Federal Service on Consumers’ Rights Protection and Human Well-Being Surveillance, 197101 Saint-Petersburg, Russia; shaieb@pasteurorg.ru (V.A.C.); antipova@pasteurorg.ru (A.Y.A.); kirichenko@pasteurorg.ru (A.D.K.); vgdedkov@yandex.ru (V.G.D.); 2 Martsinovsky Institute of Medical Parasitology, Tropical and Vector Borne Diseases, First Moscow State Medical University (Sechenov University), 119048 Moscow, Russia

**Keywords:** measles detection, real-time RT-PCR, molecular methods, outbreak

## Abstract

The severity of MeV infection has been greatly reduced by the development of a live attenuated vaccine, which has been incorporated into vaccination programs in many countries. However, poor access to health facilities, and above all, the increase in anti-vaccination movements, has prevented the achievement of sufficient vaccination coverage. In outbreak scenarios, a rapid and transportable method can improve differential diagnosis, including removing ambiguity in suspected measles cases, contacts, or a cohort. In response to the need, we have developed a new RT-qPCR-based MeV detection assay. The LOD of the developed assay was determined on different PCR machines and the higher threshold was 1–1.2 10^3^ copies/mL. The joint diagnostic sensitivity of ELISA and RT-PCR (used together) was 100%, and used combinedly, these two methods enable detection of all measles-infected persons, which is extremely important for controlling contagion and spread of infection. During the clinical validation of the assay on 200 clinical samples from measles-suspected cases using ELISA, 157 samples showed a positive result, while 163 positive cases were confirmed by the RT-qPCR assay. The concordance between the two techniques was 93%. According to our results, the real-time RT-PCR approach used in our study is more sensitive and appears to be a more promising method for measles diagnosis during early stages of the disease, likely before the rise of specific IgM antibodies detected by ELISA.

## 1. Introduction

Measles is an infectious and contagious disease caused by the measles virus, an airborne pathogen spread by inhalation of respiratory droplets. Clinical presentation of the disease is characterized by a prodrome of fever, conjunctivitis, rhinorrhea, and cough, followed by a morbilliform rash. From the middle of 20th century, vast vaccination campaigns were initiated globally, leading to a false hope of definitive measles eradication. Over the last two decades, incoming reports are indicating a progressive reemergence of measles, with multiple outbreaks registered across the globe [1] in countries with a high population index [2], with the omission bias playing a crucial a role in the increase of disease resurgence. In Western countries with a high Human Development Index, regional measles spread is related to vaccine hesitancy and under-immunized groups, including migrants from countries with poor access to health facilities [3,4,5], a fact highlighted by the WHO [6] and the European Center for Disease Prevention and Control [7]. In 2021, the WHO reported an estimated global mortality of 128,000 cases, essentially among unvaccinated or under-vaccinated children [8]. Despite the notion of measles as an already eradicated pathology, recent reports are indicating a progressive reemergence of the disease globally.

Measles virus (MeV) belongs to the *Morbillivirus* genus in the *Paramyxoviridae* family. A single-stranded, negative-sense RNA virus, MeV uses a multitude of penetration mechanisms to ensure infection of the cell using different antigenic recognition mechanisms and immune escape strategies [9].

The main risk factors, reported by a significant number of studies, are: a lack or an incompleteness of vaccination; underage for routine vaccination; migration of unvaccinated individuals; and recent contact with a measles case [10,11,12,13]. Malnutrition was also established as an essential contributor to measles infectivity in low-income countries [14]. Among comorbid factors, HIV infection was reported to increase measles susceptibility among children, but not adults [15,16].

Measles can lead to complications such as pneumonia, otitis media, thrombocytopenia, and encephalitis. In cases of malnourishment, the disease may lead to blindness, deafness, or encephalitis-associated intellectual disabilities (acute disseminated encephalomyelitis, subacute sclerosing panencephalitis) [17,18].

To overcome these epidemiological challenges, multiple laboratory detection methods were implemented during recent measles outbreaks across the globe. Currently, the mainstream WHO-recommended measles diagnostic method is ELISA-based immunoglobulin M (IgM) detection [19]. However, the IgM test detects MeV only in symptomatic individuals with late-onset symptoms, such as rash. A study investigating a measles outbreak highlighted that serum specimens collected within 4 days of initial symptoms onset were IgM-negative. However, they were simultaneously MeV RNA-positive in 82.1% of cases when analyzed by RT-qPCR [20]. The RT-PCR method allows a more accurate MeV viral load assessment in a given specimen during initiation of the infectious process and appearance of initial symptoms (fever, coryza, conjunctivitis) [21]. Earlier-diagnosed cases afford timely anti-epidemic measures and restriction of local transmission.

RNA-dependent RNA polymerase (RdRp) is an enzyme involved in replication of the viral genome, wherein RNA is synthesized from an RNA template. The enzyme and its products are key determinants of viral function and survival. Besides being a proprietary target in anti-measles therapeutics, the *RdRp* gene is also a prime target in measles molecular detection strategies. RT-qPCR targeting the *RdRp* gene can be useful during outbreak scenarios because it allows rapid detection of MeV-infected persons, increasing the number of secondary transmission cases. This method could be implemented in both stationary and mobile laboratories, particularly in low-resource settings. Such diagnostics can improve differential diagnosis, including removing ambiguity in suspected measles cases, contacts, or a cohort.

This paper describes: the development of a new MeV detection assay; clinical validation of the findings in a sample of MeV-infected patients; and evaluation of the clinical and diagnostic efficiency of several approaches. These are offered in an effort to overcome outbreak management challenges, including improvement of surveillance measures at national and international levels.

## 2. Results

### 2.1. MV AmpPS Assay Design

**Figure 1:**
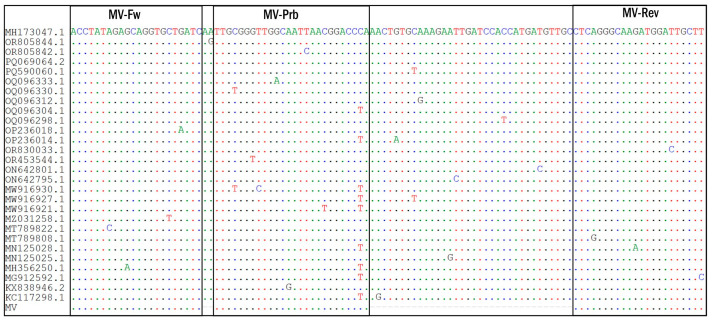
Partial nucleotide sequence alignment of MeV (*RdRp* gene). Complete sequences of MeV genotype B3 and D8 (as of December 2023) in Gen-Bank (NCBI, n = 1265) were downloaded. Among these, 544 complete *RdRp* sequences (without gaps and ambiguities of reading) were aligned to identify conserved sites. In sum, 28 unique MeV *RdRp* gene sequences with the following NCBI GenBank accession numbers are shown: MH173047.1—strain MVs/Ancona.ITA/45.17/1/[D8], OR805844.1—strain MVs/Ahmednagar.IND/48.22/233783[D8]MIBE, OR805842.1—strain MVs/Mumbai.IND/05.23/2/233874[D8]MIBE, PQ069064.2—strain MVi/Rabat.MAR/29.24/[B3], PQ590060.1—strain MVs/SaoPaulo.BRA/41.24[D8], OQ096333.1—strain MVs/Alberta.CAN/27.18[D8], OQ096330.1—strain MVs/Britih_Columbia.CAN/44.18[B3], OQ096312.1—strain MVs/British_Columbia.CAN/6.19[D8], OQ096304.1—strain MVs/British_Columbia/25.18/2[D8], OQ096298.1—strain MVs/Ontario.CAN/10.18[D8], OP236018.1—strain MVi/Pune.IND/38.13[D8], OP236014.1—strain MVi/Tumkur.Ind/06.08[D8], OR830033.1—strain MVs/Sao Paulo.BRA/02.20[D8], OR453544.1—strain MVs/Sao Paulo.BRA/239.19[D8], ON642801.1—strain MVi/Hoima.UGA/01.13 [B3], ON642795.1—strain MVi/Otuke.UGA/13.19[B3], MW916930.1—strain MVi/Krishnagiri.Ind/12.06[D8], MW916927.1—strain MVi/Anand.IND/33.15/2[D8], MW916921.1—strain MVi/Sundargarh.IND/10.14[D8], MZ031258.1—strain MVi/Marseille.FRA/27.19/6[B3], MT789822.1—strain MVs/California.USA/15.19/2[D8], MT789808.1—strain MVs/California.USA/43.19/2[B3], MN125028.1—strain MVi/Udupi.IND/7.15/7, MN125025.1—strain MVi/Kanpur.IND/16.14[D8], MH356250.1—strain MVi/Kozhikode.IND/14.10[D8], KT732240.1—strain MVs/Hull.GBR/47.12/[D8], KX838946.2—strain MVi/Calais.FRA/01.16[B3], KC117298.1—strain MVi/Vicenza.ITA/12.10/2 [D8]. Sequences of the forward (MV_Fw) and reverse (MV_Rev) primers and probes (MV_Prb) targeting this region are indicated in the frames.

The multiple alignment of the MeV genotype B3 and D8 sequences available in GenBank NCBI (n = 1265) performed at the beginning of the study (Figure 1) allowed the identification of fairly conserved regions required for the designing of the MeV-specific primers and respective probes (Table 3). Based on these data, oligonucleotide primers and fluorescent probes were designed and synthesized, and the MV AmpPS assay was developed. The developed assay includes all components required for RT-PCR. The advantage of this assay is that it allows the verification of all steps of the analysis, including extraction, reverse transcription, and PCR. In addition, by using NEC− and C− controls, the risk of false-positive results because of cross-contamination was minimized.

**Figure 2:**
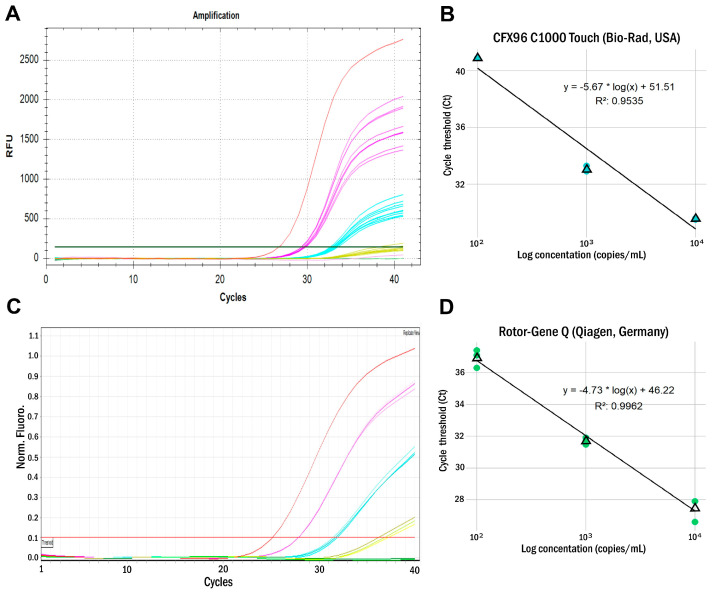
The assay LODs and assay standard curves assessed using ARCs on plate- and rotary-type instruments. Samples: red—C+, purple—ARC 10^4^ copies/mL, blue—ARC 10^3^ copies/mL, yellow—ARC 10^2^ copies/mL. The ARC 10^1^ copies/mL (pink), internal control (green), and C− (green) samples are negative. (**A**) CFX96 C1000 Touch (Bio-Rad, USA) standard curve; (**B**) LOD assay, (CFX96 C1000 Touch); (**C**) Rotor-Gene Q (Qiagen, Germany) standard curve; (**D**) LOD assay, Rotor-Gene Q.

The limit of detection (LOD) was assessed using ARC dilutions on two PCR machines featuring flatbed (Bio-Rad CFX96 C1000 Touch, USA) or rotary (Rotor-Gene Q, Qiagen, Germany) designs (Figure 2). The LOD of the assay on the CFX96 PCR plate machine, measured as the minimum dilution found in all replicates, was 10^3^ RNA copies per mL, while the LOD* measured using Probit analysis was 1.2 × 10^3^ RNA copies/mL. For the Rotor-Gene Q PCR rotary machine, the measured minimum dilution found in all replicates was above 10^2^ RNA copies/mL, while the LOD* measured using probit analysis was 2.7 × 10^2^ RNA copies/mL (Table 1).

The potential for cross-reactivity was assessed using heterologous RNA/DNA from 18 viral species belonging to 10 viral families. None of the 18 different viral species showed a positive reaction on the MeV real-time RT-PCR assay (Table 5). Consequently, the evaluated analytical specificity was 100%.

### 2.2. Clinical Evaluation of the MV AmpPS Assay

**Figure 3:**
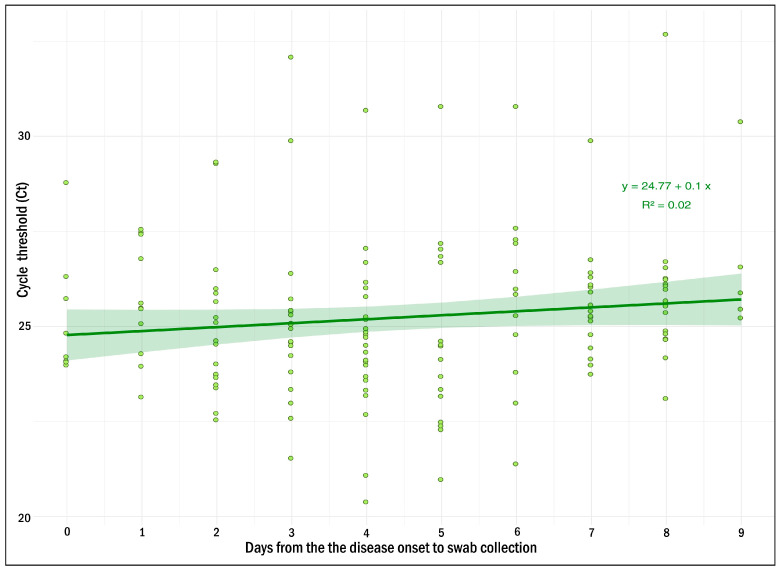
Ct value distribution of positive swab samples by collection day after disease onset.

The clinical samples (nasopharyngeal swabs, plasma) from suspected measles cases were simultaneously tested using the MV AmpPS RT-PCR assay (nasopharyngeal swabs, n = 200) and the VectoMeasles-IgM ELISA kit (Vector-Best^®^, Russia) (plasma, n = 200). The findings were: 163/200 (81.5%) matched positive (Ct values ranging from 20.4 to 32.7) (Figure 3); and 33/200 (16.5%) matched negative using both RT-PCR and ELISA methods. In addition, 13/200 (6.5%) discordant samples were observed: four ELISA-positive cases with negative RT-PCR; nine ELISA-negative cases with positive RT-PCR (Ct values ranging from 22.4 to 29.9); and one ELISA-uncertain case with positive RT-PCR (Ct = 26.8) (Table S1).

The discordant samples were retested using the single-target RT-PCR assay design [22] used at the Centers for Disease Control and Prevention (CDC, Atlanta, GA, USA) and elsewhere for MeV surveillance and case confirmation (CDC assay) with modifications related to recently observed priming mismatch variants [23]. Complete coincidence of the testing results by both real-time RT-PCR assays was observed.

In silico analysis of primers and probe sequences showed an absence of significant substitutions in the target regions of the MeV genotype B3 and D8 sequences available in NCBI GenBank (Figure 1). Thus, the MV AmpPS RT-PCR assay was found to be suitable for measles diagnostics.

## 3. Discussion

According to WHO reports, 40 million children globally did not receive routine measles vaccinations in 2021 due to the COVID-19 pandemic: 25 million missed the first dose, and 14.7 million missed the second [24,25]. Historically, the National Program for Measles Elimination in Russia (established in 2003) has greatly contributed to the eradication of the infection. However, the importation of cases originating from neighboring countries remains a problem to be solved [26].

The lifting of restrictions related to the COVID-19 pandemic and the resumption of migratory flows towards Russia have resulted in an increase in the number of imported measles cases from Central Asia. This has led to a major measles outbreak since the end of 2022, forcing a prompt reaction from health authorities to contain it. Abramov et al. reported that significant migratory flows and insufficient vaccination against measles (both in Russia and abroad) create a threat that measles virus can be imported and spread across Russia, leading to persistence nationally [27].

Traditionally, the WHO-recommended gold standard for measles diagnostics has been detection of measles-specific serum IgM antibody, mainly using the ELISA approach. With the advent of the RT-PCR methodology, and due to its cost-effectiveness, real-time RT-PCR is now a WHO newly recommended method for importation tracking and rapid diagnostics, especially in terms of commercial kits [19].

In the context of the current epidemiological situation, the Saint Petersburg Pasteur Institute has developed a new real-time RT-PCR assay for MeV detection: MV AmpPS. A summary of the developed assay is shown in Table 2.

Conditions for the real-time RT-PCR assay were initially optimized using purified RNA from modified MS2 phage particles and RNA/DNA from 18 viral species belonging to 10 viral families. For the selected primers and probe, suitable conditions for PCR with real-time detection were determined. Studies have shown that this approach can effectively differentiate MeV from closely related viruses.

The LOD of the developed assay varied on different devices. Regarding the device with a higher threshold, it was 1–1.2 × 10^3^ copies/mL (15–20 copies/reaction), which is a good indicator for diagnostic kits in the RT-PCR format with real-time detection [28,29].

Evaluation of the assay using clinical samples from measles-suspected cases showed 93% concordance between ELISA and the developed real-time RT-PCR assay. Specifically, 186 of 200 clinical samples showed the same result when tested by RT-PCR and ELISA. The obtained diagnostic sensitivity for ELISA (average) was 94.0% (95% CI: 89.26% to 97.09%). For the real-time RT-PCR assay, it was 97.6% (95% CI: 93.98% to 99.34%). These data are comparable with those described earlier [30]. Four swabs from patients with ELISA-confirmed measles were tested as negative by the assay developed, as well as by the CDC assay. This suggests that a negative test result is not a consequence of mismatches in the target region of MeV genome (the assays target different fragments of the genome). The collection date for these swabs ranged from day 1 to day 9 from disease onset. As shown in Figure 3 and Table S1, the viral load, depending on the day of the disease onset, is quite variable from sample to sample and could reach Ct values up to 24 from day 1 to the day 9 from disease onset. Therefore, the negative test result is probably not related to the virus mutations or the absence of the virus in the patient’s nasopharynx during swab collection, but to the bias during material collection or RNA extraction. Nevertheless, dependence of the viral load on the day of the disease is important for understanding in what time frame the use of real time RT-PCR is the optimal one. This issue has yet to be resolved.

Thus, the real-time RT-PCR approach is more sensitive and appears to be a more promising method for measles diagnosis. This may be the case particularly in the early stages of the disease, likely before the rise of specific IgM antibodies. Meanwhile, the joint diagnostic sensitivity of ELISA and RT-PCR (used together) was 100%. Therefore, usage of both techniques (ELISA and real-time RT-PCR) can enable detection of all measles-infected persons, which is extremely important for controlling contagion and the spread of infection. At the initial stages of the disease and in the prodromal period, the use of the RT-PCR method is more appropriate. Moreover, the method can be used not only in suspected measles cases but also in persons who have been in contact with them.

Ct values of positive swab samples allow us to conclude that the viral load in measles can vary significantly. Also, we can talk about a trend towards a decrease in viral load of swab samples over time (Figure 3). However, the viral load in the samples collected on days 7–10 from disease onset remains at a level that allows confident detection of MeV RNA by the assay developed. Thus, the optimal time frame for swabs collection could be defined in the range from 0 up to 10 days from the disease onset.

## 4. Methods and Materials

### 4.1. Development of the MV AmpPS Assay

#### 4.1.1. Identification of Conserved Sites

Complete sequences of MeV genotype B3 and D8 (as of December, 2023) in GenBank (NCBI, n = 1265) were downloaded. Among these, 544 complete *RdRp* sequences (without gaps or ambiguities of reading) were aligned to identify conserved sites. The only measles genotypes that had been reported since 2020 were B3 and D8, indicating that they were the prevalent genotypes worldwide [23].

Alignment was performed using the BioEdit 7.2.5 software package (Ibis Biosciences, Carlsbad, CA, USA). A 106-nucleotide fragment of the *RdRp* gene was selected as a target for amplification using PLOTCON (http://emboss.bioinformatics.nl/cgi-bin/emboss/plotcon, accessed on the 3 December 2023). The target corresponded to nucleotide positions 15404–15299 in the MeV reference sequence: strain MVs/Moscow.RUS/20.23/2[B3] (GenBank accession number OR290098).

The primers and probe were designed in accordance with guidelines regarding TaqMan primers and probes for RT-PCR techniques [31,32]. Primer melting temperatures were calculated using an oligonucleotide property calculator (https://bsu.bio/molbiol/oligocalc.html) [33]. In addition, an oligonucleotide property calculator and MFOLD were used to assess the thermodynamic characteristics of the probes and the probability of the appearance of secondary structures (https://www.unafold.org/mfold/applications/dna-folding-form.php, accessed on the 3 December 2023) [34]. Two nucleotides «CA» were added to the 3′ end of the MV-Prb probe sequence to form a hairpin. The rhodamine 6G (R6G) fluorescent reporter dye was covalently attached to the MeV-specific probe at the 5′ end. The black hole quencher (BHQ1) was attached at the 3′ end. The primers and probe were synthesized by Genterra PLC (Moscow, Russia). The primer and probe sequences are shown in Table 3.

#### 4.1.2. Positive and Internal Control Preparation

The assay uses several types of control samples. The control sample set is similar to that already described [35,36], and includes internal extraction control (IC), armored RNA control (ARC), negative extraction control (NEC), and PCR controls (C+, C−). Briefly, the internal extraction control and armored RNA control are modified MS2 phage particles that include either an artificial sequence (internal extraction control, IC) or a sequence of the virus to be detected (armored RNA control, ARC). Extraction control samples are added to each sample during the nucleic acid extraction step. The positive PCR control (C+) contains a mixture of plasmids containing the same regions. The negative PCR control (C−) and negative extraction control (NEC) contain ultrapure water (milli Q, Merck KGaA, Darmstadt, Germany). A 177-nucleotide MeV target fragment was synthesized de novo by a previously described two-step PCR method [37]. Phusion high-fidelity DNA polymerase (NEB, Ipswich, MA, USA) was used with the primers listed (Table 4) to create cDNA and RNA controls.

#### 4.1.3. Reaction Mixture and Amplification Conditions

Reactions were carried out in a volume of 25 μL. Optimized PCR reaction conditions were as follows: 1 µL of BioMaster Mix (Biolabmix, Novosibirsk, Russia); 12.5 µL of 2× reaction buffer (Biolabmix, Novosibirsk, Russia); 10.5 pmol of each MeV specific primer (MV-Fw, MV-Rev); 7.5 pmol of MV-probe; 10.5 pmol of internal control primers; and 7.5 pmol of internal control probe. The sample quantity used was 10 μL, and reactions were brought to 25 µL with ultrapure water (milli Q, Merck Millipore, Merck KGaA, Darmstadt, Germany). The optimized amplification regimen was the following: 50 °C 15 min; 95 °C 5 min; followed by 40 cycles (95 °C 10 s and 60 °C 30 s). Fluorescence was recorded during the 60 °C step in the HEX/yellow channel (for MeV) and FAM/green channel (for IC). The sequences of primers and probes targeting IC have been described previously [35]. The reaction was performed using the CFX96 C1000 Touch (Bio-Rad, Hercules, CA, USA) and Rotor-Gene Q (Qiagen, Hilden, Germany) PCR machines.

### 4.2. Limit of Detection

The limit of detection (LOD) was determined using a series of 10-fold dilutions of armored RNA particles (ARC, same as positive control for reverse transcription) at known concentrations. Concentrations were measured by droplet digital PCR (ddPCR). The concentrations used to determine the detection limit were 10^4^, 10^3^, 10^2^, and 10 copies of shielded RNA particles per milliliter (200, 20, 2, and 0.2 copies/reaction, respectively). Samples from each dilution (100 µL) were extracted in triplicate using the RIBO-prep kit (AmpliSens^®^, Moscow, Russia) according to the manufacturer’s instructions (elution volume 60 µL), followed by testing in the developed MV AmpPS RT-PCR assay. The LOD was set as the minimum dilution found in all replicates. Alternatively, we estimated LOD* using probit analysis.

### 4.3. Analytical Specificity

Analytical specificity was tested on a panel of viral RNA/DNA samples (solutions) representing heterologous viral strains obtained from the collection of the Saint Petersburg Pasteur Institute. Viral loads of the samples were assessed using specific real-time RT-PCR (Table 5, Figure S1).

### 4.4. Clinical Evaluation of the MV AmpPS Assay

#### 4.4.1. Sample Collection

Nasopharyngeal swabs and blood samples were collected from those suspected to have measles (N = 200), specifically those admitted to hospitals in Saint Petersburg or the Leningrad Region from 3 May to 20 September 2024. Patient ages ranged from four months to 63 years. Women accounted for 46.5% and men for 53.5%.

Blood samples were collected 3–5 days after rash manifestation, while nasopharyngeal swabs were collected 1–3 days after rash manifestation (Table S1, Supplementary Materials).

Epidemiological and clinical information for each patient was collected using a standardized form. To ensure the confidentiality of personal patient data, each clinical record was assigned an individual number, which was subsequently used to label the tubes containing samples for molecular–biological and serological studies. Samples were delivered to the Saint Petersburg Pasteur Institute and kept for further studies.

Nasopharyngeal swabs were collected in 500 µL of special transport medium or phosphate-buffered saline (pH 7.0). Blood samples were taken into vacutainers with EDTA and processed by centrifugation (3000× *g* for 5 min). Plasma was separated from cellular elements and transferred into plastic tubes. All samples were stored at −80 °C until further analysis.

#### 4.4.2. Enzyme-Linked Immunosorbent Assay

The presence of specific IgM antibodies against the MeV antigen was determined using the VectoMeasles-IgM ELISA kit (Vector-Best^®^, Novosibirsk, Russia) according to the manufacturer’s instructions.

#### 4.4.3. RNA Extraction

Total nucleic acids from nasopharyngeal swabs were extracted and purified using the RIBO-prep kit (AmpliSens^®^, Moscow, Russia) according to the manufacturer’s instructions (elution volume 100 µL) and stored at −80 °C until molecular analysis.

## 5. Conclusions

This manuscript reports the development and evaluation of the MV AmpPS real-time RT-PCR assay for measles virus detection targeting an *RdRp* gene fragment. The assay contains all of the necessary components to perform the analysis, including internal extraction control (IC), positive reverse-transcription control (ARC), negative control of extraction (NCE), positive PCR control (C+), and negative PCR control (C−). The advantage of this assay is that it allows the verification of all steps of the analysis, including extraction, reverse transcription, and PCR.

Our results revealed that the LOD of the assay is in the range of 1–1.2 × 10^3^ RNA copies/mL on the CFX96 PCR plate machine. On the Rotor-Gene Q PCR rotary machine, the LOD is in the range of 1—2.7 × 10^2^ RNA copies/mL (10–27 RNA copies/reaction). The LOD of the assay described is comparable with previously described MeV RT-PCR assays (22 copies/reaction and 10 copies/reaction, respectively) [22,23] as well as with RT-RPA CRISPR/Cas12a (120 copies/reaction) and RT-RPA-CRISPR-LFD assays (6.25 copies/reaction) [30]. Thus, this assay can be effectively used for detection of the reemergent B3 and D8 genotype strains [22,38,39] in the course of diagnostics and epidemiological surveillance of measles.

## Figures and Tables

**Table 1:** Threshold cycles (Ct) of the HEX/yellow channel (ARC dilution) at various concentrations.

ARC Concentration (Copies/mL)	CFX96 C1000 Touch (Bio-Rad, USA)				Rotor-Gene Q (Qiagen, Germany)			
	Replicate, C_t_ Values				Replicate, C_t_ Values			
	1	2	3	Median	1	2	3	Median
10^4^	29.6	29.5	29.6	29.6	27.9	27.9	27.9	27.9
10^3^	33.3	32.9	33.0	33.1	31.5	31.9	31.7	31.7
10^2^	neg.	neg.	40.9	neg.	37.1	37.4	36.3	36.9
10^1^	neg.	neg.	neg.	neg.	neg.	neg.	neg.	neg.
LOD (copies/mL)	10^3^				10^2^			
LOD* (copies/mL)	1.2 × 10^3^				2.7 × 10^2^			

Note: LOD*—limit of detection measured using probit analysis; neg.—negative.

**Table 2:** Summary of the MV AmpPS assay.

Parameter	Real-Time RT-PCR Assay
Reagent components	MeV super mix, RT-PCR enzyme mix, 2× RT-PCR buffer, armored internal control (IC), armored positive RNA control (ARC+), positive PCR control (C+), negative PCR control (C−), and negative extraction control (NEC)
Virus detected	*Morbillivirus hominis* (MeV)
Genetic target	*RdRp* gene
Real-time PCR platform	CFX96 C1000 Touch (Bio-Rad, USA), Rotor-Gene Q (Qiagen, Germany)
Nucleic acid extraction required	yes
Suitable specimens for testing	nasopharyngeal swabs
Sensitivity	1–1.2 × 10^3^ copies/mL (CFX96 C1000 Touch), 1–2.7 × 10^2^ copies/mL (Rotor-Gene Q)
Duration of the analysis (excluding extraction time)	90 min

**Table 3:** List of primers and probes used.

Primer or Probe	Sequence (5′→3′)	Reference Sequence Nucleotide Position (GenBank OR290098)	5′–3′ Modification	Amplicon Length (bp)
MV-Fw	ACCTATAgAGCAggTgCTgATC	15299–15320	none	106
MV-Rev	AAgCAATCCATCTTGCCCTgAg	15383–15404	none	
MV-Prb	TTgCgggTTggCAATTAACggACC***CA*** *	15323–15348	R6G—BHQ1	-
IC-Fw	CCggATTgCgTATCTCCggACT	none	none	122
IC-Rev	CACggCggCATCTCTATCACgA	none	none	
IC-Prb1	CTAgCTgggCgTCAggAATCCCAgg	none	FAM-BHQ1	-

* The two nucleotides added to the 3′ end of the MV-Prb sequence are marked in bold italic font.

**Table 4:** Oligonucleotides used for de novo MeV cDNA synthesis by two-step PCR.

Name	Sequence (5′-3′)
MEV1	AGTTATTAGAGGTGATATCAACCCTACTCTGAAAAAACTTACACCTATAGAGCAGGTG
MEV2	GTTTGGGTCCGTTAATTGCCAACCCGCAATTGATCAGCACCTGCTCTATAGGTGTAAGTT
MEV3	GGCAATTAACGGACCCAAACTGTGCAAAGAATTGATCCACCATGATGTTGCCTCAGGGCA
MEV4	AACTCCCTGTAGAGGATGAGTATAGAATTAAGCAATCCATCTTGCCCTGAGGCAACATCA

**Table 5:** List of viral species used in this study.

Species	Acronym	Family	Genus	Type of Nucleic Acid	RT-PCR Kit	Ct Value	MV AmpS Assay
Influenza A/H1N3	FLUAV (H1N3)	*Orthomyxoviridae*	*Alphainfluenzavirus*	RNA	AmpliSens^®^ Influenza virus A-type-FRT PCR	18.4	negative
Influenza A/H3N2	FLUAV (H3N2)	*Orthomyxoviridae*	*Alphainfluenzavirus*	RNA		17.9	negative
Influenza B	FLUBV	*Orthomyxoviridae*	*Betainfluenzavirus*	RNA	AmpliSens^®^ Influenza virus A/B-FRT PCR	23.6	negative
Human parainfluenza virus type 1	HPIV-1	*Paramyxoviridae*	*Rubulavirus*	RNA	AmpliSens^®^ ARVI-screen-FRT	20.4	negative
Human parainfluenza virus type 4b	HPIV-4b	*Paramyxoviridae*	*Rubulavirus*	RNA	AmpliSens^®^ ARVI-screen-FRT	23.8	negative
Human rhinovirus B (Type 17)	HRV-B	*Picornaviridae*	*Enterovirus*	RNA	AmpliSens^®^ ARVI-screen-FRT	24.4	negative
Human adenovirus type 6	HAdV-6	*Adenoviridae*	*Mastadenovirus*	DNA	AmpliSens^®^ All screen-FRT	19.8	negative
Severe acute respiratory syndrome coronavirus 2	SARS-CoV-2	*Coronaviridae*	*Betacoronavirus*	RNA	COVID-19Amp (St. Petersburg Pasteur Institute) [35]	17.3	negative
Rubella virus	RUBV	*Matonaviridae*	*Rubivirus*	RNA	NA	22.7	negative
Respiratory syncytial virus type B1	RSV B1	*Pneumoviridae*	*Orthopneumovirus*	RNA	AmpliSens^®^ ARVI-screen-FRT	21.9	negative
Human parechovirus type 1	HPeV-1	*Picornaviridae*	*Parechovirus*	RNA	NA	-	negative
Human alphaherpesvirus 1	HSV-1	*Herpesviridae*	*Alphaherpesvirus*	DNA	AmpliSens^®^ HSV I, II-FRT	21.8	negative
Human Rotavirus A	RVA	*Reoviridae*	*Rotavirus*	RNA	AmpliSens^®^ Rotavirus/Norovirus/Astrovirus-FRT PCR	19.9	negative
Echovirus 4	ECHOV-4	*Picornaviridae*	*Enterovirus*	RNA	NA	-	negative
Human Cytomegalovirus 5	HCMV-5	*Herpesviridae*	Cytomegalovirus	DNA	AmpliSens^®^ CMV-FRT PCR	19.9	negative
Human parvovirus B19	B19	*Parvoviridae*	Erythroparvovirus	DNA	AmpliSens^®^ Parvovirus B19-FRT PCR	22.1	negative
Human Coxsackievirus B1	CV-B1	*Picornaviridae*	Enterovirus	RNA	NA	-	negative
Mumps orthorubulavirus	MuV	*Paramyxoviridae*	Orthorubulavirus	RNA	NA	-	negative

## Data Availability

The original contributions presented in the study are included in the article. Further inquiries can be directed to the corresponding author.

## Supplementary Materials

The following supporting information can be downloaded at https://www.mdpi.com/article/10.3390/ijms26051801/s1.
