# Supplementary material for: Development and Evaluation of a New Measles Detection Assay Using Real-Time RT-PCR
Source: Int J Mol Sci. 2025 Feb 20;26(5):1801. doi: 10.3390/ijms26051801 (PMC11898969; doi:10.3390/ijms26051801)
Supplement: Supplementary file 1 [file ijms-26-01801-s001.zip › ijms-3448094-supplementary.pdf]

Supplementary

Table S1. Samples from measles suspected cases used in this study.

N	ID	Sex	Date of birth	Date of disease onset	Date of rash manifestation	Date of swab collection	Date of blood collection	VectoMeasles-IgM ELISA kit	MV AmpPS assay, Ct value
1	2024.1313	male	07.02.2024	12.06.2024	14.06.2024	16.06.2024	17.06.2024	positive	24.3
2	2024.1212	female	05.04.2023	24.05.2024	28.05.2024	30.05.2024	31.05.2024	negative	negative
3	2024.1185	male	26.01.2024	20.05.2024	22.05.2024	22.05.2024	25.05.2024	positive	29.3
**4**	**2024.1132**	**male**	**12.08.2007**	**18.05.2024**	**18.05.2024**	**19.05.2024**	**21.05.2024**	**uncertain**	**26.8**
5	2024.1348	male	07.04.1985	09.06.2024	16.06.2024	18.06.2024.	20.06.2024	positive	30.4
6	2024.1121	female	10.08.2019	09.05.2024	14.05.2024	16.05.2024	17.05.2024	negative	negative
7	2024.1145	male	26.07.2017	13.05.2024	18.05.2024	18.05.2024	21.05.2024	positive	22.4
8	2024.1251	male	29.06.2007	29.05.2024	03.06.2024	04.06.2024	06.06.2024	positive	27.2
9	2024.1310	female	02.06.1967	09.06.2024	11.06.2024	13.06.2024	14.06.2024	positive	23.7
10	2024.1322	female	07.10.2023	12.06.2024	15.06.2024	15.06.2024.	18.06.2024	negative	negative
11	2024.1274	male	16.06.2022	02.06.2024	06.06.2024	06.06.2024	09.06.2024	positive	22.7
12	2024.1273	female	28.07.2015	02.06.2024	06.06.2024	06.06.2024	09.06.2024	positive	20.4
13	2024.1174	female	01.03.2020	19.05.2024	22.05.2024	23.05.2024	25.05.2024	positive	25.8
14	2024.1146	female	26.05.2011	17.05.2024	17.05.2024	20.05.2024	22.05.2024	positive	32.1
15	2024.1333	male	07.08.2022	13.06.2024	17.06.2024	19.06.2024.	20.06.2024	positive	30.8
16	2024.1147	male	05.07.2010	15.05.2024	18.05.2024	18.05.2024	21.05.2024	positive	23.0
**17**	**2024.1120**	**female**	**28.01.2013**	**14.05.2024**	**16.05.2024**	**18.05.2024**	**19.05.2024**	**positive**	**negative**
18	2024.1350	female	09.09.2022	14.06.2024	19.06.2024	20.06.2024.	22.06.2024	positive	24.8
19	2024.1196	female	02.02.2023	24.05.2024	26.05.2024	27.05.2024	29.05.2024	positive	25.4
20	2024.1239	male	06.07.2007	31.05.2024	02.06.2024	04.06.2024	05.06.2024	positive	23.6
21	2024.1317	male	14.04.2014	08.06.2024	13.06.2024	13.06.2024	17.06.2024	positive	23.7
22	2024.1219	male	16.05.2023	27.05.2024	30.05.2024	01.06.2024	03.06.2024	positive	24.5
23	2024.1355	male	06.03.2007	17.06.2024	21.06.2024	23.06.2024.	25.06.2024	positive	27.6
24	2024.1282	female	26.01.1970	03.06.2024	07.06.2024	10.06.2024	10.06.2024	positive	24.0
25	2024.1115	male	30.07.2017	12.05.2024	16.05.2024	17.05.2024	19.05.2024	positive	30.8
26	2024.1178	female	04.04.2020	21.05.2024	24.05.2024	25.05.2024	27.05.2024	positive	26.7
27	2024.1252	female	28.11.2023	31.05.2024	03.06.2024	03.06.2024	06.06.2024	positive	22.6
28	2024.1311	male	01.07.1975	09.06.2024	09.06.2024	13.06.2024	14.06.2024	negative	negative
29	2024.1347	male	01.09.1995	16.06.2024	18.06.2024	19.06.2024.	21.06.2024	positive	29.9
30	2024.1352	male	14.10.1954	18.06.2024	20.06.2024	21.06.2024.	24.06.2024	negative	negative
31	2024.1314	female	25.09.2010	09.06.2024	13.06.2024	14.06.2024.	17.06.2024	positive	22.5
**32**	**2024.1270**	**female**	**12.08.1968**	**01.06.2024**	**04.06.2024**	**08.06.2024**	**08.06.2024**	**negative**	**29.9**
33	2024.1163	male	09.08.2023	17.05.2024	21.05.2024	21.05.2024	24.05.2024	positive	21.1
34	2024.1323	female	17.01.2022	12.06.2024	14.06.2024	16.06.2024.	18.06.2024	positive	30.7
35	2024.1357	male	03.01.1978	18.06.2024	22.06.2024	24.06.2024.	25.06.2024	positive	21.4
36	2024.1162	female	30.08.2023	17.05.2024	21.05.2024	21.05.2024	24.05.2024	positive	25.2
37	2024.1287	male	03.09.1981	05.06.2024	08.06.2024	10.06.2024	11.06.2024	positive	26.7
38	2024.1184	male	08.06.2023	18.05.2024	22.05.2024	23.05.2024	27.05.2024	negative	negative
39	2024.1268	male	10.05.1961	31.05.2024	01.06.2024	08.06.2024	08.06.2024	positive	32.7
40	2024.1195	female	27.05.2023	24.05.2024	26.05.2024	27.05.2024	29.05.2024	positive	22.6
41	2024.1285	male	18.04.2008	04.06.2024	08.06.2024	09.06.2024	11.06.2024	positive	22.3
42	2024.1281	male	20.07.2000	04.06.2024	07.06.2024	08.06.2024	10.06.2024	negative	negative
**43**	**2024.1140**	**male**	**07.01.2021**	**19.05.2024**	**19.05.2024**	**19.05.2024**	**22.05.2024**	**negative**	**28.8**
44	2024.1133	female	16.04.2017	15.05.2024	19.05.2024	18.05.2024	21.05.2024	positive	25.1
45	2024.1321	female	06.07.2023	10.06.2024	14.06.2024	16.06.2024.	18.06.2024	positive	26.0
46	2024.1271	male	22.05.2023	01.06.2024	05.06.2024	05.06.2024	08.06.2024	positive	23.2
47	2024.1186	male	28.10.2022	19.05.2024	22.05.2024	23.05.2024	25.05.2024	positive	24.8
**48**	**2024.1269**	**female**	**05.07.1975**	**05.06.2024**	**05.06.2024**	**08.06.2024**	**08.06.2024**	**negative**	**24.6**
**49**	**2024.1197**	**male**	**28.12.1975**	**21.05.2024**	**26.05.2024**	**29.05.2024**	**29.05.2024**	**positive**	**negative**
50	2024.1170	female	17.03.1987	16.05.2024	19.05.2024	22.05.2024	23.05.2024	positive	27.3
**51**	**2024.1267**	**male**	**19.04.1983**	**31.05.2024**	**03.06.2024**	**08.06.2024**	**08.06.2024**	**negative**	**24.9**
52	2024.1284	male	24.03.2023	03.06.2024	08.06.2024	08.06.2024	11.06.2024	positive	22.4
53	2024.1335	female	19.03.2021	12.06.2024	16.06.2024	17.06.2024	19.06.2024	negative	negative
54	2024.1343	female	04.07.2015	11.06.2024	17.06.2024	17.06.2024	20.06.2024	positive	23.0
55	2024.1327	male	25.04.2006	11.06.2024	14.06.2024	18.06.2024	18.06.2024	positive	24.0

56	2024.1355	male	06.03.2007	17.06.2024	21.06.2024	23.06.2024.	25.06.2024	positive	27.6
57	2024.1357	male	03.01.1978	18.06.2024	22.06.2024	24.06.2024	25.06.2024	positive	21.4
58	2024.1370	male	03.06.2017	17.08.2024	20.08.2024	18.08.2024	21.08.2024	positive	25.6
59	2024.1425	female	30.11.2015	09.08.2024	13.08.2024	NA	14.08.2024	positive	24.3
60	2024.1442	male	22.10.1991	02.08.2024	08.08.2024	06.08.2024	10.08.2024	positive	25.7
61	2024.1667	male	28.08.2016	14.09.2024	18.09.2024	17.09.2024	20.09.2024	positive	27.3
62	2024.1773	female	25.09.2020	12.07.2024	16.07.2024	15.07.2024	18.07.2024	positive	23.5
63	2024.1812	male	02.11.2017	10.08.2024	11.08.2024	NA	14.08.2024	positive	30.7
64	2024.1894	male	01.04.2022	04.08.2024	08.08.2024	07.08.2024	08.08.2024	positive	26.5
65	2024.1941	male	02.09.2021	11.07.2024	13.07.2024	13.07.2024	14.07.2024	positive	26.1
66	2024.2095	female	02.03.2008	23.07.2024	27.07.2024	27.07.2024	27.07.2024	positive	24.8
67	2024.2152	female	17.04.2024	21.08.2024	21.08.2024	21.08.2024	22.08.2024	positive	25.9
68	2024.2245	female	04.01.1994	06.09.2024	10.09.2024	10.09.2024	12.09.2024	negative	negative
69	2024.2250	male	28.09.1989	24.08.2024	27.08.2024	27.08.2024	27.08.2024	positive	26.5
70	2024.2292	male	11.02.1970	26.07.2024	30.07.2024	30.07.2024	01.08.2024	positive	24.8
71	2024.2364	male	15.02.1967	09.09.2024	12.09.2024	10.09.2024	12.09.2024	positive	27.2
72	2024.2469	female	02.08.2000	06.06.2024	10.06.2024	10.06.2024	10.06.2024	positive	25.2
73	2024.2813	female	12.02.2024	27.06.2024	30.06.2024	01.07.2024	03.07.2024	positive	23.7
74	2024.2818	female	29.07.1979	28.07.2024	02.08.2024	02.07.2024	02.08.2024	positive	24.0
75	2024.2843	female	11.04.2008	18.07.2024	23.07.2024	23.07.2024	24.07.2024	positive	25.9
76	2024.2874	female	08.10.1973	15.08.2024	19.08.2024	19.08.2024	21.08.2024	negative	negative
77	2024.2901	female	03.11.2013	07.06.2024	10.06.2024	07.06.2024	10.06.2024	positive	24.0
78	2024.3085	male	11.02.2009	23.06.2024	25.06.2024	26.06.2024	28.06.2024	positive	21.6
79	2024.3108	male	05.11.2023	14.07.2024	17.07.2024	14.07.2024	17.07.2024	negative	negative
80	2024.3245	female	28.01.2015	02.07.2024	03.07.2024	04.07.2024	06.07.2024	positive	29.3
81	2024.3283	male	10.12.2010	20.07.2024	23.07.2024	20.07.2024	23.07.2024	positive	24.1
82	2024.3309	male	13.05.1982	14.07.2024	16.07.2024	14.07.2024	16.07.2024	negative	negative
83	2024.3310	male	18.08.1976	04.08.2024	05.08.2024	04.08.2024	05.08.2024	positive	26.5
84	2024.3354	male	06.09.1982	12.07.2024	13.07.2024	12.07.2024	13.07.2024	positive	26.1
85	2024.3610	female	10.09.1975	13.08.2024	14.08.2024	13.08.2024	14.08.2024	positive	23.1
86	2024.3705	female	01.10.2022	14.06.2024	17.06.2024	19.06.2024	20.06.2024	positive	23.2
87	2024.3727	male	02.09.2007	21.07.2024	23.07.2024	21.07.2024	23.07.2024	positive	26.4
88	2024.3833	male	04.02.1991	17.07.2024	19.07.2024	17.07.2024	19.07.2024	negative	negative
89	2024.3967	male	01.10.1974	16.09.2024	17.09.2024	16.09.2024	17.09.2024	positive	25.1
90	2024.4025	male	17.08.2002	15.07.2024	17.07.2024	15.07.2024	17.07.2024	positive	26.2
91	2024.4051	female	24.05.2024	20.08.2024	21.08.2024	20.08.2024	21.08.2024	positive	25.2
92	2024.4165	male	05.09.1997	17.09.2024	18.09.2024	17.09.2024	18.09.2024	negative	negative
93	2024.4169	female	20.08.1992	22.07.2024	23.07.2024	22.07.2024	23.07.2024	positive	24.9
94	2024.4218	female	21.11.2022	17.07.2024	19.07.2024	17.07.2024	19.07.2024	positive	24.2
95	2024.4284	male	04.07.1997	11.09.2024	12.09.2024	11.09.2024	12.09.2024	positive	23.8
96	2024.4350	female	14.09.2009	06.08.2024	08.08.2024	06.08.2024	08.08.2024	positive	26.1
97	2024.4370	female	15.09.1962	22.07.2024	23.07.2024	22.07.2024	23.07.2024	positive	26.0
98	2024.4416	male	19.03.1983	21.07.2024	22.07.2024	21.07.2024	22.07.2024	positive	27.0
99	2024.4440	male	13.11.2023	11.07.2024	13.07.2024	11.07.2024	13.07.2024	positive	25.8
100	2024.4467	female	13.01.2012	15.09.2024	17.09.2024	15.09.2024	17.09.2024	negative	negative
101	2024.4489	male	01.04.2065	16.06.2024	19.06.2024	16.06.2024	19.06.2024	positive	27.3
102	2024.4517	female	28.03.2006	05.08.2024	05.08.2024	02.08.2024	05.08.2024	positive	23.9
103	2024.4543	female	05.01.1992	30.06.2024	02.07.2024	30.06.2024	02.07.2024	positive	25.3
104	2024.4611	female	19.05.2002	17.09.2024	18.09.2024	17.09.2024	18.09.2024	positive	23.0
105	2024.4612	female	07.01.2019	30.07.2024	02.08.2024	30.07.2024	02.08.2024	positive	25.6
106	2024.4695	female	23.03.2016	16.09.2024	17.09.2024	16.09.2024	17.09.2024	positive	25.4
107	2024.5080	male	25.07.2089	07.09.2024	08.09.2024	06.09.2024	08.09.2024	positive	25.2
108	2024.5124	male	03.07.1979	17.08.2024	21.08.2024	18.08.2024	21.08.2024	positive	25.2
109	2024.5148	female	30.12.1998	12.07.2024	17.07.2024	15.07.2024	17.07.2024	positive	25.4
110	2024.5168	male	29.12.1971	26.07.2024	30.07.2024	28.07.2024	30.07.2024	positive	24.6
**111**	**2024.5241**	**female**	**19.01.2024**	**10.09.2024**	**12.09.2024**	**10.09.2024**	**12.09.2024**	**negative**	**24.2**
112	2024.5312	male	03.09.2001	16.07.2024	18.07.2024	17.07.2024	18.07.2024	positive	26.2
113	2024.5318	female	09.07.2023	06.08.2024	08.08.2024	06.08.2024	08.08.2024	negative	negative
114	2024.5362	male	28.01.2023	18.07.2024	19.07.2024	18.07.2024	19.07.2024	positive	26.3
**115**	**2024.5408**	**male**	**25.04.1986**	**08.08.2024**	**08.08.2024**	**06.08.2024**	**09.08.2024**	**negative**	**23.1**
116	2024.5441	male	14.04.2020	13.07.2024	13.07.2024	12.07.2024	13.07.2024	negative	negative
117	2024.5560	female	03.07.2010	03.05.2024	03.05.2024	02.05.2024	03.05.2024	positive	24.8
**118**	**2024.5675**	**male**	**05.04.1996**	**03.08.2024**	**04.08.2024**	**08.08.2024**	**09.08.2024**	**positive**	**negative**
119	2024.5692	female	06.11.2004	17.07.2024	18.07.2024	17.07.2024	18.07.2024	positive	25.7
120	2024.5694	male	03.11.1961	30.07.2024	30.07.2024	29.07.2024	30.07.2024	positive	23.6
121	2024.5819	female	28.04.1989	27.08.2024	27.08.2024	26.08.2024	27.08.2024	positive	24.3
122	2024.5846	male	31.07.2023	14.08.2024	14.08.2024	11.08.2024	14.08.2024	positive	25.5
123	2024.5858	male	06.06.1987	19.07.2024	19.07.2024	17.07.2024	19.07.2024	positive	25.1

124	2024.5877	male	04.10.2001	18.07.2024	18.07.2024	16.07.2024	18.07.2024	negative	negative
125	2024.6042	male	12.10.2007	13.07.2024	13.07.2024	11.07.2024	13.07.2024	negative	negative
126	2024.6067	male	04.11.1986	18.09.2024	18.09.2024	16.09.2024	18.09.2024	positive	24.0
127	2024.6087	male	22.01.2014	30.07.2024	30.07.2024	28.07.2024	30.07.2024	positive	24.6
128	2024.6132	male	26.01.2021	16.07.2024	16.07.2024	13.07.2024	16.07.2024	positive	25.0
129	2024.6190	male	07.05.2000	13.07.2024	13.07.2024	12.07.2024	13.07.2024	positive	23.5
130	2024.6210	female	02.11.2006	22.07.2024	22.07.2024	20.07.2024	22.07.2024	positive	26.9
131	2024.6303	male	15.08.2010	02.08.2024	02.08.2024	01.08.2024	02.08.2024	negative	negative
132	2024.6336	male	18.12.2020	08.08.2024	08.08.2024	05.08.2024	08.08.2024	positive	24.4
133	2024.6474	male	13.08.1970	18.09.2024	18.09.2024	17.09.2024	18.09.2024	positive	25.4
134	2024.6484	female	22.02.1974	18.07.2024	18.07.2024	16.07.2024	18.07.2024	positive	25.6
135	2024.6523	female	01.09.2020	18.07.2024	18.07.2024	17.07.2024	18.07.2024	positive	24.8
136	2024.6595	female	16.06.2004	02.08.2024	02.08.2024	30.07.2024	02.08.2024	positive	26.3
137	2024.6666	female	07.03.1984	16.07.2024	16.07.2024	15.07.2024	16.07.2024	positive	24.5
138	2024.6705	female	03.05.1988	17.09.2024	17.09.2024	16.09.2024	17.09.2024	positive	24.4
139	2024.6741	male	19.11.2007	03.07.2024	07.07.2024	07.07.2024	10.07.2024	positive	23.3
140	2024.6809	male	31.08.1982	18.07.2024	18.07.2024	16.07.2024	18.07.2024	positive	26.2
141	2024.6906	male	18.07.1980	27.08.2024	27.08.2024	25.08.2024	27.08.2024	positive	24.9
142	2024.6924	male	06.01.2024	13.07.2024	13.07.2024	10.07.2024	13.07.2024	positive	27.1
143	2024.6986	female	01.07.1991	30.07.2024	30.07.2024	27.07.2024	30.07.2024	positive	24.8
144	2024.7045	female	03.08.2023	13.07.2024	13.07.2024	11.07.2024	13.07.2024	positive	28.0
145	2024.7122	male	09.06.1996	17.09.2024	17.09.2024	15.09.2024	17.09.2024	positive	25.8
146	2024.7149	female	03.04.2010	20.09.2024	20.09.2024	17.09.2024	20.09.2024	positive	25.0
147	2024.7344	male	05.09.2012	05.08.2024	05.08.2024	03.08.2024	05.08.2024	positive	26.3
148	2024.7366	male	17.06.2008	19.07.2024	19.07.2024	17.07.2024	19.07.2024	positive	25.2
149	2024.7401	male	15.01.2016	22.07.2024	22.07.2024	21.07.2024	22.07.2024	positive	26.9
150	2024.7448	female	11.06.1998	02.08.2024	02.08.2024	01.08.2024	02.08.2024	negative	negative
151	2024.7452	female	01.03.2018	27.06.2024	27.06.2024	26.06.2024	27.06.2024	positive	26.3
152	2024.7534	male	17.02.1991	05.08.2024	05.08.2024	04.08.2024	05.08.2024	positive	26.1
153	2024.7566	female	27.05.2024	12.09.2024	12.09.2024	10.09.2024	12.09.2024	positive	26.9
**154**	**2024.7655**	**female**	**13.09.2020**	**30.07.2024**	**30.07.2024**	**28.07.2024**	**30.07.2024**	**negative**	**24.5**
155	2024.7709	male	08.11.1984	16.07.2024	16.07.2024	14.07.2024	16.07.2024	negative	negative
156	2024.7850	female	01.08.1992	13.08.2024	13.08.2024	12.08.2024	13.08.2024	positive	25.4
157	2024.7874	female	05.07.2021	19.07.2024	19.07.2024	17.07.2024	19.07.2024	negative	negative
158	2024.7884	female	08.09.1984	13.07.2024	13.07.2024	11.07.2024	13.07.2024	negative	negative
159	2024.7934	female	07.02.2022	27.08.2024	27.08.2024	26.08.2024	27.08.2024	negative	negative
160	2024.8021	female	17.12.2023	18.09.2024	18.09.2024	16.09.2024	18.09.2024	positive	26.2
161	2024.8072	male	01.12.2023	04.09.2024	11.09.2024	12.09.2024	14.09.2024	positive	23.1
162	2024.8077	male	12.03.1991	13.07.2024	13.07.2024	10.07.2024	13.07.2024	negative	negative
163	2024.8082	male	22.04.2002	27.08.2024	27.08.2024	26.08.2024	27.08.2024	positive	24.2
164	2024.8105	female	02.06.1985	18.07.2024	18.07.2024	16.07.2024	18.07.2024	positive	23.4
165	2024.8173	male	12.09.1985	27.08.2024	27.08.2024	26.08.2024	27.08.2024	positive	24.1
166	2024.8181	female	14.04.2005	14.08.2024	14.08.2024	12.08.2024	14.08.2024	positive	25.6
167	2024.8270	female	22.09.2021	22.07.2024	22.07.2024	19.07.2024	22.07.2024	positive	25.8
168	2024.8291	female	24.07.1964	16.07.2024	16.07.2024	14.07.2024	16.07.2024	positive	26.8
**169**	**2024.8338**	**male**	**03.04.1998**	**25.08.2024**	**27.08.2024**	**25.08.2024**	**27.08.2024**	**negative**	**22.4**
**170**	**2024.8473**	**male**	**14.03.1973**	**25.08.2024**	**27.08.2024**	**25.08.2024**	**27.08.2024**	**positive**	**negative**
171	2024.8487	male	11.11.2010	21.08.2024	21.08.2024	18.08.2024	21.08.2024	positive	27.2
172	2024.8548	female	03.12.2022	17.09.2024	17.09.2024	16.09.2024	17.09.2024	negative	negative
173	2024.8565	male	09.08.2016	17.07.2024	17.07.2024	16.07.2024	17.07.2024	negative	negative
174	2024.8569	female	03.10.2020	14.08.2024	14.08.2024	11.08.2024	14.08.2024	positive	25.8
175	2024.8680	male	15.08.2019	08.09.2024	08.09.2024	06.09.2024	08.09.2024	positive	25.9
176	2024.8777	female	02.11.1987	30.07.2024	30.07.2024	29.07.2024	30.07.2024	positive	27.0
177	2024.8783	male	19.04.1981	08.09.2024	08.09.2024	05.09.2024	08.09.2024	positive	25.2
178	2024.8812	female	12.05.1994	12.09.2024	12.09.2024	11.09.2024	12.09.2024	negative	negative
179	2024.8834	female	10.11.2022	11.09.2024	12.09.2024	15.09.2024	18.09.2024	negative	negative
180	2024.8896	male	02.06.1983	31.08.2024	31.08.2024	29.08.2024	31.08.2024	positive	25.0
181	2024.8992	female	30.11.2015	10.08.2024	11.08.2024	NA	14.08.2024	positive	25.0
182	2024.8995	female	04.12.1970	22.06.2024	22.06.2024	20.06.2024	22.06.2024	positive	25.8
183	2024.9029	female	23.08.2007	18.07.2024	18.07.2024	17.07.2024	18.07.2024	positive	26.9
184	2024.9076	female	05.10.19654	28.06.2024	28.06.2024	27.06.2024	28.06.2024	positive	27.7
185	2024.9134	male	09.08.1978	29.06.2024	03.07.2024	04.07.2024	06.07.2024	positive	25.7
186	2024.9181	female	11.10.1970	18.09.2024	18.09.2024	17.09.2024	18.09.2024	positive	21.0
187	2024.9250	male	23.06.1993	17.07.2024	17.07.2024	14.07.2024	17.07.2024	positive	24.1
**188**	**2024.9363**	**male**	**04.08.2022**	**14.08.2024**	**16.08.2024**	**17.08.2024**	**19.08.2024**	**negative**	**24.7**
189	2024.9377	female	25.06.2003	23.07.2024	23.07.2024	21.07.2024	23.07.2024	positive	25.1
190	2024.9393	male	04.05.1967	16.07.2024	16.07.2024	15.07.2024	16.07.2024	positive	23.8
191	2024.9405	female	12.08.2022	05.08.2024	05.08.2024	04.08.2024	05.08.2024	negative	negative

192	2024.9413	male	21.05.2017	14.08.2024	14.08.2024	12.08.2024	14.08.2024	positive	24.8
193	2024.9425	male	15.07.2016	07.08.2024	07.08.2024	04.08.2024	07.08.2024	positive	26.5
194	2024.9493	male	02.01.1976	17.07.2024	17.07.2024	16.07.2024	17.07.2024	positive	23.4
195	2024.9549	male	05.02.2003	19.07.2024	19.07.2024	18.07.2024	19.07.2024	negative	negative
196	2024.9664	female	14.11.1972	21.08.2024	21.08.2024	18.08.2024	21.08.2024	negative	negative
197	2024.9755	female	07.06.1963	12.09.2024	12.09.2024	10.09.2024	12.09.2024	positive	25.0
198	2024.9759	male	09.10.1970	17.07.2024	17.07.2024	15.07.2024	17.07.2024	positive	25.3
199	2024.9763	female	09.07.1958	13.07.2024	13.07.2024	11.07.2024	13.07.2024	positive	23.4
200	2024.9999	female	31.03.1971	19.07.2024	19.07.2024	18.07.2024	19.07.2024	positive	25.2

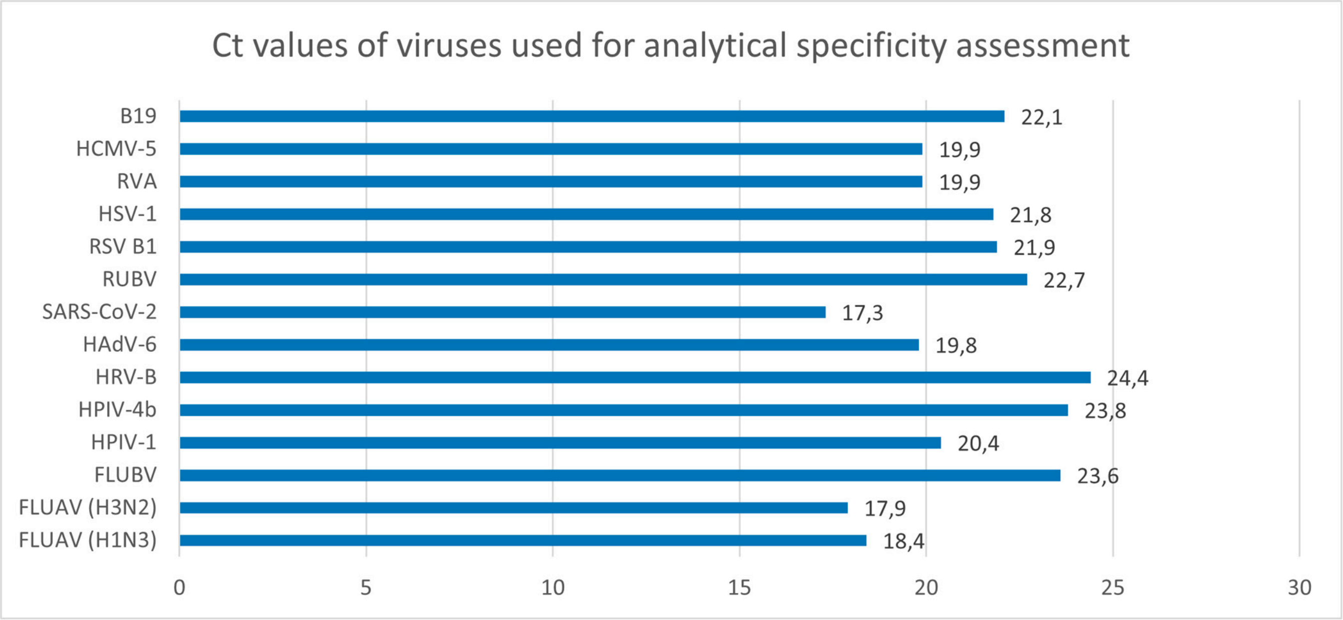

Figure S1. Ct values of viruses used for analytical specificity assessment
